# Spinal Cord Müllerian-Like Cyst at the T12 Vertebral Level: A Case Report and Literature Review

**DOI:** 10.7759/cureus.80169

**Published:** 2025-03-06

**Authors:** Takaaki Uto, Satoshi Kato, Noriaki Yokogawa, Masafumi Kawai, Satoru Demura

**Affiliations:** 1 Department of Orthopaedic Surgery, Graduate School of Medical Sciences, Kanazawa University, Kanazawa, JPN

**Keywords:** case report, intradural extramedullary, müllerian cyst, spinal cord cyst, t12 vertebra

## Abstract

Müllerian cysts are rare developmental anomalies, typically found in the pelvis, with extrapelvic occurrences reported but spinal cord involvement being exceedingly rare. We present the first reported case of a Müllerian-like cyst located on the surface of the spinal cord at the T12 vertebral level. A 49-year-old woman presented with low back and right leg pain, including electric shock-like sensations during sneezing. Physical examination revealed sensory deficits in the right L5 dermatome, and MRI demonstrated an intradural extramedullary cystic lesion at T12, appearing isointense to cerebrospinal fluid on all sequences, with a subtle surface irregularity. A T12 recapping T-saw laminoplasty was performed for surgical excision, and a subtle surface defect on the spinal cord was identified intraoperatively. Pathological examination confirmed the diagnosis of a Müllerian-like cyst. This case highlights the importance of considering Müllerian-like cysts in the differential diagnosis of intradural spinal cysts, even at unusual locations, and demonstrates that careful review of imaging findings, including subtle surface irregularities, can aid in accurate diagnosis and surgical planning. Complete surgical resection is the treatment of choice, and the prognosis is generally excellent.

## Introduction

Müllerian cysts are rare developmental anomalies that originate from remnants of the Müllerian duct and are typically pelvic [1,2]. Extrapelvic occurrences of Müllerian cysts, including those in the retroperitoneum and posterior mediastinum, have been reported [3-6]; however, spinal cord involvement is exceptionally rare. Although various intradural spinal lesions, including arachnoid cysts [7,8], neurenteric cysts [9], bronchogenic cysts [10], epidermoid cysts [11], and even spinal cord tuberculomas [12] or syphilitic meningomyelitis [13], have been described, a Müllerian-like cyst in this location is exceedingly rare. A comprehensive literature search using databases such as PubMed, Google Scholar, Scopus, and Web of Science revealed no prior reports of Müllerian-like cysts originating on the surface of the spinal cord. This rarity complicates preoperative diagnosis, often leading to misidentification as more common lesions such as schwannomas [7], which are rare even in their purely cystic form in the thoracic spine [14]. Such misidentification can lead to unnecessary or inappropriate management, underscoring the importance of accurate preoperative diagnosis. We present a Müllerian-like cyst on the surface of the spinal cord at the T12 vertebral level, to our knowledge, the first reported case, emphasizing the diagnostic challenges and the importance of meticulous imaging review and surgical planning.

## Case presentation

**Figure 1:**
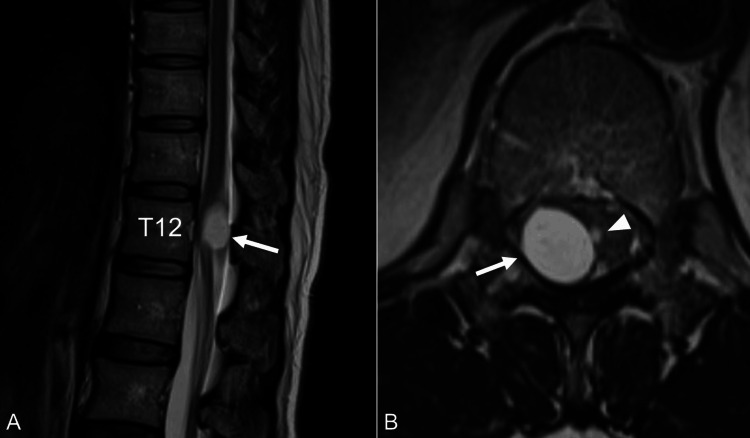
Preoperative magnetic resonance imaging (MRI) of the thoracic spine. (A) Sagittal T2-weighted image showing an intradural extramedullary cyst at the T12 vertebral level (arrow). (B) Axial T2-weighted image demonstrating the cyst (arrow) and a subtle surface irregularity (arrowhead) on the right side of the spinal cord surface.

**Figure 2:**
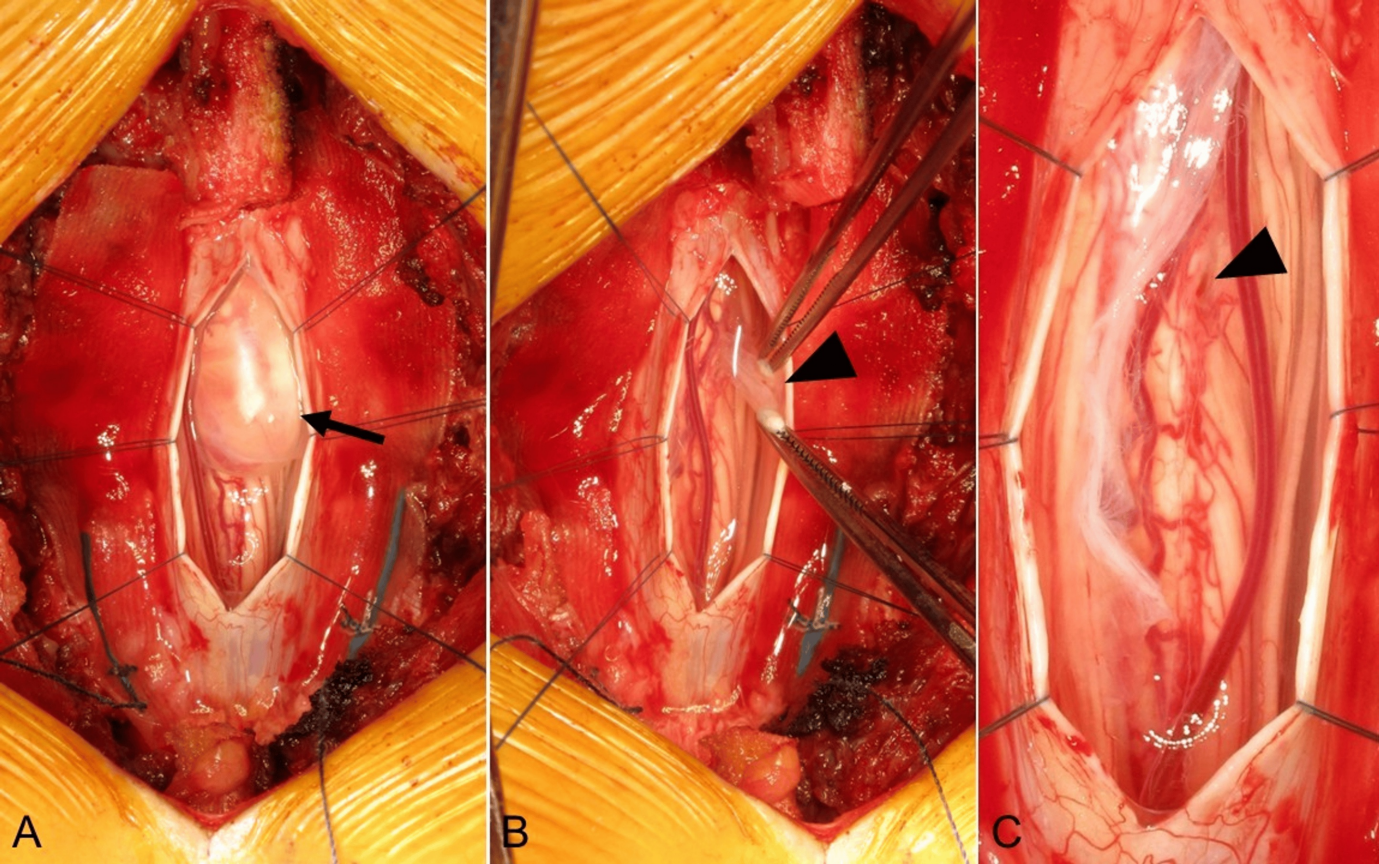
Intraoperative findings. (A) The cystic lesion (arrow) is identified after dural and arachnoid incision. (B) The cyst membrane is observed to be ruptured (arrowhead). (C) After the membrane is excised, a minor surface defect is noted on the dorsal surface of the spinal cord (arrowhead).

**Figure 3:**
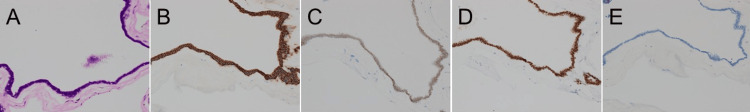
Histopathological examination and immunohistochemical findings of the cyst. (A) Hematoxylin and eosin (H&E) staining showing a cyst wall lined by a single layer of cuboidal to columnar epithelium. (B) Immunohistochemistry for cytokeratin AE1/AE3 demonstrating strong positivity, confirming the epithelial nature of the lining cells. (C) Estrogen receptor (ER) positivity supporting Müllerian differentiation. (D) PAX8 positivity further indicating Müllerian origin. (E) S-100 negativity, helping to exclude nerve sheath-related lesions.

A 49-year-old woman presented with a several-month history of low back pain radiating to her right leg and foot. She also experienced electric shock-like sensations in the same distribution triggered by sneezing. Her past medical history was unremarkable, with no history of spinal trauma, surgery, or known congenital anomalies. On physical examination, she exhibited pain and numbness in the right L5 dermatome. Patellar and Achilles tendon reflexes were diminished on the right side. The remainder of the neurological examination was normal, with no signs of myelopathy or upper motor neuron involvement. Magnetic resonance imaging (MRI) of the thoracic spine revealed an intradural extramedullary cystic lesion on the right side at the T12 vertebral level. The lesion demonstrated low signal intensity on T1-weighted images, high signal intensity on T2-weighted and short tau inversion recovery (STIR) images, well-defined margins, and no gadolinium enhancement (Figure 1). The cyst contents appeared isointense to cerebrospinal fluid (CSF) on all sequences. Given the symptomatic nature of the lesion and the diagnostic uncertainty, surgical intervention was planned. Intraoperative neuromonitoring using motor evoked potentials (MEPs) and somatosensory evoked potentials (SEPs) was employed. A T12 recapping T-saw laminoplasty [15] was performed to access the spinal canal. After midline dural and arachnoid incisions, a clear cystic lesion was identified, seemingly originating on the surface of the right dorsal spinal cord (Figure 2A). During careful manipulation, the cyst wall ruptured, releasing clear fluid, but the cyst membrane remained intact (Figure 2B). The cyst was meticulously excised near the spinal cord surface, where a subtle surface defect was noted (Figure 2C). MEPs and SEPs remained stable throughout the procedure. The dura was closed primarily, and the T12 lamina was restored using the recapping technique. The surgery was uneventful. Postoperative pathological examination revealed a cyst wall lined by a single layer of cuboidal to columnar epithelium, with occasional ciliated cells (Figure 3A). Immunohistochemical staining demonstrated strong positivity for cytokeratin AE1/AE3 (Figure 3B), estrogen receptor (ER) (Figure 3C), and PAX8 (Figure 3D), supporting a Müllerian-like origin. The cells were negative for S-100 protein (Figure 3E), helping to exclude nerve sheath tumors such as schwannomas. It is important to note that while the epithelial lining and immunohistochemical profile strongly suggested a Müllerian origin, endometrial-type stroma, a key component for confirming a true Müllerian cyst, was absent in the cyst wall. Therefore, the term "Müllerian-like cyst" was used to reflect the partial Müllerian characteristics while acknowledging the absence of definitive Müllerian stroma. Based on these findings, the final diagnosis was a Müllerian-like cyst of the spinal cord. At the two-year follow-up, the patient reported significant improvement in her symptoms. Mild residual numbness in the right leg persisted, but the electric shock-like pain triggered by sneezing had resolved. No evidence of cyst recurrence was observed on follow-up imaging, which included MRI of the thoracic spine.

## Discussion

This case report presents the first documented instance of a Müllerian-like cyst located on the surface of the spinal cord at the T12 vertebral level. Müllerian cysts typically arise from remnants of the Müllerian duct and are most commonly found in the pelvis [1,2]. While extrapelvic locations such as the retroperitoneum and posterior mediastinum have been reported [3-5], spinal cord involvement is exceedingly rare.

The location of this cyst significantly expands the differential diagnosis of intradural extramedullary cystic lesions. The primary differential diagnoses include arachnoid cysts, schwannomas, neurenteric cysts, bronchogenic cysts, and epidermoid cysts [7-11]. Less common possibilities include infectious etiologies, such as spinal cord tuberculomas [12] or syphilitic meningomyelitis [13]. While spinal schwannomas and arachnoid cysts are more prevalent, even purely cystic thoracic schwannomas are rare [14], highlighting the diagnostic challenges.

In our case, the preoperative MRI findings were suggestive of a benign cystic lesion, but a definitive diagnosis was difficult. The cyst appeared isointense to CSF on all sequences, which is a common feature of arachnoid cysts. However, the retrospectively identified subtle surface irregularity on the axial T2-weighted image (Figure 1B, arrowhead) proved to be a crucial clue. This finding, confirmed intraoperatively, confirmed a surface attachment between the cyst and the spinal cord, making a simple arachnoid cyst less likely. This emphasizes the importance of meticulous review of imaging studies, even for seemingly minor findings [16].

The definitive diagnosis of a Müllerian-like cyst was established by histopathological and immunohistochemical examination. The presence of a single layer of cuboidal to columnar epithelium with occasional cilia, along with strong positivity for cytokeratin AE1/AE3, ER, and PAX8, and negativity for S-100, confirmed the Müllerian origin and excluded other possibilities, such as schwannomas. While the epithelial lining with cilia and the immunohistochemical profile (positive for ER and PAX8) strongly suggested a Müllerian origin, it is important to acknowledge that endometrial-type stroma, a key component for confirming a true Müllerian cyst, was absent in the cyst wall. Therefore, we deliberately chose the term "Müllerian-like cyst" to reflect the partial Müllerian characteristics while acknowledging the absence of definitive Müllerian stroma. It is also possible to consider the current case as a broader category of migration disorders, given the unusual location of the lesion. Further research may be needed to better understand the specific mechanisms.

Complete surgical resection is the gold standard treatment for symptomatic intradural spinal cysts, providing both definitive diagnosis and preventing recurrence [17,18]. In our case, T12 recapping T-saw laminoplasty allowed for safe and complete excision of the cyst with minimal disruption of surrounding neural structures. Intraoperative neuromonitoring was crucial for ensuring the safety of the procedure. While this is the first reported case of a Müllerian-like cyst in this specific location, complete surgical resection of symptomatic intradural extramedullary cystic lesions, when feasible, is often associated with a favorable prognosis [19]. Furthermore, the prognosis for patients with completely resected Müllerian cysts in other locations (e.g., pelvis, retroperitoneum) is generally excellent [6]. Based on these analogies, and given the complete resection achieved in our case, a favorable long-term outcome was expected. However, long-term follow-up is still recommended to monitor for any potential recurrence, although the risk is considered low [20]. Aspiration is generally not recommended due to high recurrence risk and potential complications, especially given the close proximity to the spinal cord [9]. Adjuvant therapies like radiation or chemotherapy are generally not indicated for benign, completely resected Müllerian-like cysts.

## Conclusions

This case report presents the first documented instance of a Müllerian-like cyst arising on the surface of the spinal cord, specifically at the T12 vertebral level. This previously unreported location for a Müllerian cyst expands the known spectrum of Müllerian duct anomalies and highlights the importance of considering this entity in the differential diagnosis of intradural extramedullary spinal cysts, even when imaging findings appear typical of more common lesions. The subtle surface irregularity identified on preoperative MRI, and confirmed intraoperatively as a defect on the spinal cord surface, proved to be a valuable clue for accurate diagnosis. Complete surgical resection via T12 recapping T-saw laminoplasty was successful, leading to significant symptom improvement and no evidence of recurrence at two-year follow-up. This case underscores the critical role of meticulous radiological interpretation and pathological examination in the diagnosis and management of rare spinal pathologies, and it emphasizes that complete surgical excision offers an excellent prognosis for patients with symptomatic, surgically accessible Müllerian-like cysts of the spinal cord.
